# Comparative Analysis of the Mitochondrial Genomes of Callitettixini Spittlebugs (Hemiptera: Cercopidae) Confirms the Overall High Evolutionary Speed of the AT-Rich Region but Reveals the Presence of Short Conservative Elements at the Tribal Level

**DOI:** 10.1371/journal.pone.0109140

**Published:** 2014-10-06

**Authors:** Jie Liu, Cuiping Bu, Benjamin Wipfler, Aiping Liang

**Affiliations:** 1 Key Laboratory of Zoological Systematics and Evolution, Institute of Zoology, Chinese Academy of Sciences, Chaoyang District, Beijing, P. R. China; 2 Graduate University of the Chinese Academy of Sciences, Shijingshan District, Beijing, P. R. China; 3 Jiangsu Key Laboratory for Eco-Agricultural Biotechnology around Hongze Lake, Huaiyin Normal University, Huaian, Jiangsu Province, P. R. China; University of Lausanne, Switzerland

## Abstract

The present study compares the mitochondrial genomes of five species of the spittlebug tribe Callitettixini (Hemiptera: Cercopoidea: Cercopidae) from eastern Asia. All genomes of the five species sequenced are circular double-stranded DNA molecules and range from 15,222 to 15,637 bp in length. They contain 22 tRNA genes, 13 protein coding genes (PCGs) and 2 rRNA genes and share the putative ancestral gene arrangement of insects. The PCGs show an extreme bias of nucleotide and amino acid composition. Significant differences of the substitution rates among the different genes as well as the different codon position of each PCG are revealed by the comparative evolutionary analyses. The substitution speeds of the first and second codon position of different PCGs are negatively correlated with their GC content. Among the five species, the AT-rich region features great differences in length and pattern and generally shows a 2–5 times higher substitution rate than the fastest PCG in the mitochondrial genome, *atp8*. Despite the significant variability in length, short conservative segments were identified in the AT-rich region within Callitettixini, although absent from the other groups of the spittlebug superfamily Cercopoidea.

## Introduction

Callitettixini (Hemiptera: Cercopoidea: Cercopidae) is a small tribe currently containing eleven described species in three genera distributed in China and Southeast Asia [1]. Commonly known as froghoppers or spittlebugs, species of the Cercopoidea are characterized by their extremely powerful jumping ability [2], [3] and the habit of covering themselves with foamy saliva-like masses during their nymphal stages. The foams are secreted by a specialized bellow-like tube structure on the ventral surface of the abdomen of the nymphs and the foam itself is formed by mucopolysaccharides and proteins secreted by the glandular segment of the Malpighian tubules mixing with air [4]. Spittlebugs are strict plant feeders and many species exhibit strong preferences for nitrogen-fixing plants [5], [6], while some species are grass pests which can cause severe economic damage [7]. Currently the superfamily Cercopoidea is classified into five families, Cercopidae, Aphrophoridae, Machaerotidae, Clastopteridae and Epipygidae. An inter-familiar relationship, Machaerotidae + (Clastopteridae + (Cercopidae + (Aphrophoridae + Epipygidae))) was proposed by Cryan [8] and Cryan and Svenson [9], based on analysis of nuclear and mitochondrial genes.

Mitochondria are key organelles of eukaryotic cell with multiple functions including oxidative phosphorylation [10] and apoptosis [11]. Typical animal mitochondrial genomes (mitogenomes) contain 37 genes: 13 protein coding genes (PCGs), 22 tRNA genes and 2 rRNA genes. Additionally, mitochondria harbor a non-coding region commonly referred to as the control or AT-rich region due to its high content in adenine (A) and thymine (T), which is assumed to have essential regulatory functions for transcription and replication [12]. The mitogenome has many properties of heredity and evolution that differ from those of the nuclear genomes, such as maternal inheritance, lack of substantial intermolecular recombination [13]. Thus far, complete mitogenomic sequences have been used mostly for analyzing phylogenetic relationships of higher taxa [14], [15]. Hua *et al.* analyzed the family-level relationships of the Pentatomomorpha (Heteroptera) based on mitogenomes [16]. In addition to nucleotide sequences, gene arrangements can sometimes provide phylogenetic information [17]. However, in most studied insect species the gene arrangement is highly conservative and follows the putative ancestral state [18], [19]. According to the previously published data however, there are some cases of gene rearrangements in mitogenomes of Hemipteran insects. So far, these have been reported in Sternorrhyncha [20], Fulgoroidea [21] and Heteroptera [11], [22]. Gene rearrangements did not show any correlations with rates of nucleotide substitution [16].

Insect mitogenomes usually show a high proportion of nucleotides A and T, and their AT-rich region usually contains tandem repeat sequences, polythymine stretches and stem-loop secondary structures [12], [23], [24]. The AT-rich region is also called the control region for its biological function of replication [24]. However, with the increase in insect mitogenome data, it has been realized that conservation in length, nucleotide sequence and organization patterns of the AT-rich region is not universal [16], [25]. Difficulty in aligning sequences of the AT-rich region from different species is common [16], and subsequently, the evolutionary speed (viz. the nucleotide substitution rate) of this genomic region is always difficult to calculate. Analyses of the evolutionary characteristics of the AT-region at a reasonable taxonomic range are needed in order to give an insight into evolutionary mechanisms behind the great variation that is observed.

The rate of publication of mitochondrial genome data is regularly increasing. To date, more than fifty complete or near-complete mitochondrial genomes of hemipteran insects are available on GenBank. However, only three of these are Cicadomorphan mitogenomes, with two species of Cercopoidea, *Philaenus spumarius* (Aphrophoridae) [26] and *Paphnutius ruficeps* (Cercopidae) [27] and one species of the Membracidae, *Homoladisca vitripennis* (NC_006899).

To explore the tribe-level evolutionary characteristics of the spittlebug's mitogenomes, we sequenced the complete mitogenomes of five representative species of the tribe Callitettixini (Hemiptera: Cercopoidea: Cercopidae), two belonging to the genus *Abidama* and three to the genus *Callitettix*. The aims of the present study are to 1) explore the composition characteristics of the mitogenomes of the Callitettixini; 2) describe the evolutionary divergence of the mitogenomes at both tribe and genus levels; 3) investigate the phylogenetic relationships among the five species of Callitettixini based on the new mitogenome data and 4) compare and explore the possible evolutionary mechanisms of the most divergent segment, the AT-rich region.

## Material and Methods

### Taxon sampling

Table 1 shows the specimens studied in the present work. They were preserved in 100% ethanol and stored at −80°C in the Molecular Laboratory of the Cicada Group at the Institute of Zoology (IOZ), Chinese Academy of Sciences (CAS), Beijing, China.

**Table 1:** General information about the mitogenomes of the five Callitettixini species studied.

Species	Location*	Size (bp)	AT%	AT skew	GC skew	Accession
*A. contigua*	Henan	15613	76.3	0.216	−0.249	JX844626
*A. product*	Guizhou	15227	77.4	0.204	−0.233	GQ337955
*C. biformis*	Yunnan	15222	76.8	0.203	−0.264	JX844627
*C. braconoides*	Yunnan	15637	77.2	0.189	−0.214	JX844628
*C. versicolor*	Henan	15374	75.8	0.230	−0.286	EU725832
Average	-	15415	76.7	0.208	−0.249	-

*: the names of the Chinese Provinces where the specimens were collected.

### Laboratory procedures

Total genomic DNA was extracted from the thoracic muscles, legs or heads. The remaining body parts were preserved in 95% ethanol and stored at −80°C. All samples used in this work are deposited in the Zoological Museum of the Institute of Zoology, Chinese Academy of Sciences, Beijing, China. The standard phenol-chloroform extraction protocol (Molecular Cloning III) was used for the genomic DNA extraction process. The final total genomic DNA was dissolved in the TE buffer (pH 8.0) and stored at 4°C.

The PCR amplifications were conducted with 1–2 µl of genomic DNA, 0.5U Taq polymerase (Tiangen Biotechn CO., Ltd., Beijing), 1 µl 10 µM primers, 2.0 µl 2.5 mM dNTPs (Tiangen), and 2.5 µl 10× Taq buffer. Water was added to retrieve a volume of 25 µl for all samples.

The amplification reaction was performed on a MyCycler thermal cycler (Bio-Rad). The temperature cycling was set for 5 minutes with an initial denaturation at 94°C, followed by 32–36 cycles of denaturation at 94°C for 30 seconds, annealing at 50°C for 40 seconds and elongation at 72°C for 2–4 minutes (The exact time was determined according to the length of the PCR product). The amplification ended with an additional elongation for 5 minutes at 72°C. The primers used in PCR were taken from Simon *et al.* [28], [29] or newly designed based on sequences already obtained in this study (all the primers are listed in Table S1).

The PCR products were checked using gel electrophoresis (gel of 1% agarose, staining with Ethidium bromide). In most cases, the gels contained one single and clear target band under UV light (312 nm). The target bands were purified with Agarose Gel DNA Purification Kit (Tiangen) and sequenced directly with corresponding PCR primers. When the direct sequencing of the PCR product did not yield successful results, the target bands were cloned into pBS-T Vector (Tiangen) for sequencing. The sequencing was conducted using a PE/ABI 377 automated sequencer with the ABI PRISM BigDye Terminator Cycle Sequencing v 2.0 Ready Reaction Kit (PE Biosystems) following the manufacturers protocols.

### Sequence assembly, annotation and analyses

Sequencing reliability was confirmed by reading and checking the chromatograms in CHROMAS V2.3 (Technelysium, Tewantin, Queensland, Australia). The sequence proofreading and genome assembly was performed using Codon Code aligner (CodonCode Corporation, Dedham, MA). PCGs and rRNA genes were identified by aligning the sequences with the mitogenome of *Philaenus spumarius* (Hemiptera: Cercopoidea: Aphrophoridae) and other published insect mitogenomes from GenBank. The termini of the rRNA genes were identified by alignment with homologs (such as *P. spumarius* and *Drosophila melanogaster*). The tRNA genes were identified by online analyses provided by the tRNAScan-SE server (http://www.genetics.wustl.edu/eddy/tRNAscan-SE/) [30]. The tRNAs that could not be recognized by tRNAScan-SE were identified by alignment with the mitogenome of *P. spumarius*. The secondary structures of the tRNAs were inferred according to Steinberg and Cedergren [31]. The putative secondary structure folds of the AT-rich regions were inferred via online analysis using the DNA mfold web server (http://www.bioinfo.rpi.edu/applications/mfold/) [32] under default parameters apart from the folding temperature which was set to 25°C. The calculations of nucleotide composition and codon usage were conducted with MEGA 4 [33]. Dot-plot comparison of sequences for the purpose of detecting repeat regions was conducted using BioEdit [34]. MEGA 4.0 and DnaSP 5.0 [35] were used to calculate basic statistical information on the dataset. The saturation level of the sequences was tested by comparing uncorrected p-distances with the distances calculated by the Kimura two-parameter (K2p) model [36]. The calculation of pairwise distances was done with MEGA 4.0 and the scatter plot graphics were used to visualize the results.

### Phylogenetic reconstruction

Complete sequences of each gene were used in the phylogenetic analyses except for the stop codons of the PCGs. All PCGs were aligned based on amino acid sequences with MEGA using the ClustalX criterion [37]. The rRNAs and tRNAs were aligned with MUSCLE [38] under default settings. The alignments were manually checked and corrected. The subsequent alignments were concatenated for phylogenetic reconstruction. The general reversible model (GTR+I+G) with parameters optimized by Modeltest version 3.7 [39] were used. Maximum Likelihood (ML) analyses were conducted with Garli [40] while Bayesian analyses were performed with MrBayes version 3.1.2 [41] under the following parameters: four runs of 2,000,000 generations and 25% burn-in. Additional analyses were also performed with the third codon positions excluded. Maximum Parsimony (MP) analyses for all approaches were conducted in PAUP4b10 [42]. Bootstrap tests [43] were conducted with 1000 replications under the ML and MP criterions.

## Results and Discussions

In total the complete mitochondrial genomes of five Callitettixini species were sequenced for the present study. All sequence data were submitted to the GenBank. Table 1 provides basic information as well as accession IDs.

### Genome organization and nucleotide bias

The sequence data shows that each of the five mitogenomes is a circular double-stranded DNA molecule containing 37 genes (2 rRNAs, 22 tRNAs and 13 PCGs), as commonly found among metazoans [41]. The major-strand (J-strand) encodes 23 genes while the remaining 14 genes are located on the minor-strand (N-strand). All the five mitogenomes show no differences in the gene order compared with the putative ancestral states in insects [19], as with all other studied Cicadomorphan species including the spittlebugs [26], [27].

**Figure 1:**
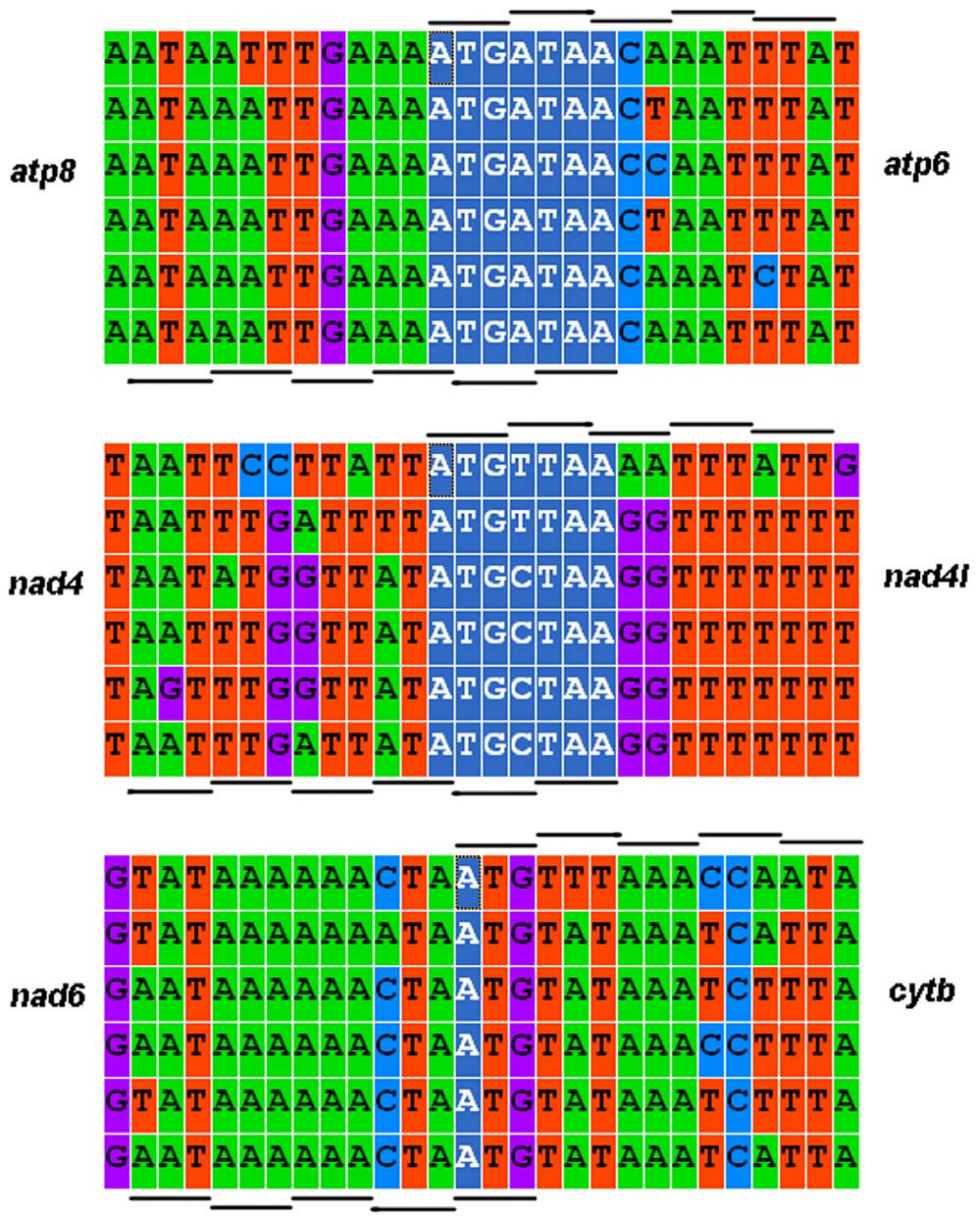
Nucleotide sequences of the overlapping areas between the protein coding genes in the mitochondrial genomes of six species of Cercopoidea (including five Callitettixini species and *Philaenus spumarius*). The short lines indicate the reading frame of the genes and the white characters show the overlapping nucleotides.

The mitogenomes of the five Callitettixini species show a high efficiency in nucleotide usage, with only very short non-coding sequences between the genes found. Overlapping nucleotides are commonly found between genes, some being conserved among different species (such as the overlapping areas between nad4/nad4L and atp6/atp8 as shown in Figure 1). In contrast to the nuclear genomes, the coding areas constitute most of the mitogenome. Except for short (<10 bp) intergenic spacers, the AT-rich region covers most of the non-coding area.

The entirety of the genomes of the five Callitettixini species show an extreme AT bias, as observed in the published data of other insects [44]. The inner environmental conditions within the mitochondria, such as the abundance of adenine caused by the high concentration of ATP [10], the high concentration of reactive oxygen species (ROS) which tends to promote the conversion from G:C to A:T or T:A, the low efficiency of the mitochondrial DNA repair system, and the requirement of maintaining translation efficiency are hypothesized to be the reasons for the high content of A and T in mitochondrial genomes [45], [46], [47].

### Protein coding genes (PCGs)

In total six kinds of triplet initiation codons (ATN, GTG and TTG) are identified in the mitogenomes of the five Callitettixini species. Thereby ATG is the most common start codon. Non-triplet start codons composed of more than 3bp have been reported in other insects [48], but are not observed in the species of the present work. Other studied hemipterans such as *Paphnutius ruficeps* (Cercopidae) also rely on 3 bp-start codons, so the present study confirms this potential apomorphy for the Cercopidae [24].

Many of the PCGs do not have complete stop codons but rather a single T or TA acting as an incomplete stop codon. These are found whenever a tRNA gene is located at the 3′ end of a PCG. Complete stop codons however are observed in the four PCGs that are not adjacent to tRNA at their 3′ includingATP synthase F0 subunit 8 gene (*atp8*), ATP synthase F0 subunit 6 gene (*atp6*), *NADH dehydrogenase subunit 4L gene* (nad4l) and NADH dehydrogenase subunit 6 (*nad6*). As suggested for other hemipteran species, we consider the stop codon as the cleavage point between the PCG and tRNA [16], [21]. The complete stop codon TAA may be generated by adding a poly(A) tail to the 3′ end of the mRNA after the transcription processes and the cleavage of the tRNA precursors [49].

**Figure 2:**
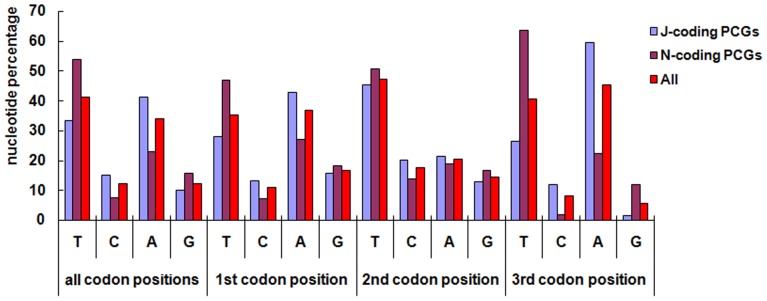
Nucleotide compositions of the codon positions of the PCGs.

The average nucleotide composition of the PCGs of the mitogenomes of five Callitettixini species is shown in Figure 2. The extreme AT nucleotide bias is observed at all of the three codon positions. However, the first and third codon positions of the J-chain PCGs contain more A than T, while the second has more T than A. In the N-Strain, positions one and three have more T than A. Similar to the J-strain, the situation in the second position is reversed (Figure 2 and Table 2). In total, T is the most common nucleotide at second codon positions (avg. 47.5%) while the proportions of the other nucleotides are more or less equal. In general, the most frequently observed fourfold degenerate codon is NNA, while NNG is the rarest (Figure 2). The same situation has been observed in other hemipterans [16], [21]. This feature may be correlated with the nucleotide bias between AT and GC (Figure 2) in mitogenomes [43].

**Table 2:** Average nucleotide composition and biases of the coding genes and AT-rich region of the mitogenomes of the five Callitettixini species.

	Pos	Chain	T(%)	C(%)	A(%)	G(%)	A+T(%)	AT-skew#	GC-skew#
PCG	1	J	28.1	13.2	42.9	15.8	73.0	0.21	0.09
		N	47.0	7.4	27.1	18.4	74.1	−0.27	0.43
	2	J	45.3	20.3	21.4	13.0	66.7	−0.36	−0.22
		N	50.6	13.8	18.9	16.7	69.5	−0.46	0.10
	3	J	26.5	12.1	59.6	1.7	86.1	0.38	−0.75
		N	62.2	2.0	24.8	11.0	87.0	−0.43	0.69
tRNAs		J	35.6	9.5	44.4	10.4	80.0	0.11	0.05
		N	35.0	14.1	43.4	7.5	78.4	0.11	0.31
rRNAs		J	-	-	-	-	-	-	-
		N	49.7	7.0	30.0	13.3	79.7	−0.25	0.31
AT-rich region*		J	38.0	10.9	43.6	7.5	81.6	0.07	0.18
Total genome*		J	29.9	14.7	46.5	8.8	76.4	0.22	0.29

*: represented with the J strand.

# : AT-skew  =  (A-T)/(A+T), GC-skew  =  (G - C)/(G+C).

**Figure 3:**
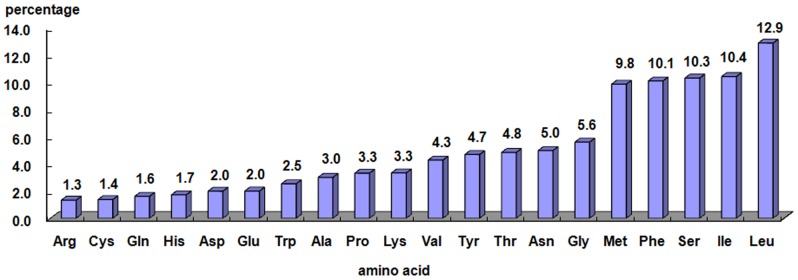
Average Amino acid frequencies of proteins coded by the mitogenomes of the five Callitettixini species given in percentage.

In the mitochondria, there are 20 amino acids which correspond to 22 tRNAs and 62 codons. Thus, each tRNA must recognize at least two types of codon. However, the biases of codon usage and amino acid composition are very strong. The translation efficiency is not affected by the non-correspondence between the amount of tRNA gene copies and the amino acid bias [17]. Figure 3 shows the amino acid composition of the proteins coded by the mitogenome. Notably, the five most common amino acids (Leu, Ile, Ser, Phe and Met) make up more than 50% of the amino acids in sum. In contrast to remaining amino acids, these five amino acids have codons with more A and T (U). It is apparent that this bias of amino acids is correlated with the bias of the nucleotides in mitogenomes [50].

### tRNA and rRNA genes

Most of the tRNA genes could be identified using tRNAScane-SE. The tRNA genes and their secondary structures that could not be detected with tRNAScane-SE were identified through alignment with other known insect tRNA genes. We here present the predicted cloverleaf folds of the 22 tRNAs of the mitogenome of *Abidama producta* as an example (Figure S1). All of the tRNAs could be folded into standard cloverleaf structures with an aminoacyl arm of 7 base pairs and anticodon arm of 5 base pairs. However, the TψC and the DHU arms which have aberrant loops and very short stems vary in length within the different tRNAs. A similar feature of secondary structure has been observed in the other two spittlebugs *Philaenus spumarius* [26] and *Paphnutius ruficeps* [27]. The conservatism of length in the aminoacyl and anticodon arm may indicate that even small changes in length will be detrimental to the functions of the tRNAs. Non-Watson-Crick matches (such as G-U and G-A) are common in cloverleaf structures and observed in the mitogenomes of other animals [51], though it is unknown whether these aberrant tRNAs still maintain their normal functions. The accumulation of these mutations may be caused by the absence of a recombination process capable of ‘fixing’ such errors [52]. However, there are reports of an RNA editing process which may ‘repair’ the mismatched acceptor stem [53], and in some cases, special nuclear tRNAs are found to penetrate into the mitochondria [54], [55].

Each of the mitogenomes of the five Callitettixini species has two rRNA genes as found in other insects: the *LR* (16S rRNA) located between tRNA-Val and tRNA-Leu (TAG) and the *SR* (12S rRNA) between the tRNA-Leu and the AT-rich region.

### Intergenic space and overlapping regions

The total length of the six available spittlebug mitogenomes (including the five Callitettixini species and *P. spumarius*) ranges between 15222 bp and 16324 bp (Table 1), which is similar to other Cicadomorphan species [26], [27]. The lengths of the coding areas (tRNAs, rRNAs, and PCGs) show a more narrow range (14451 bp to 14562 bp, including *P. spumarius*). Thus the variation among the mitogenome size is mainly caused by differences in the non-coding regions. The major non-coding region is the AT-rich region which is located between srRNA and tRNA-Ile. In general, the mitogenomes show high nucleotide efficiency in gene coding, with several genes overlapping with neighbors [43]. The three overlapping areas between the PCGs (*atp8* and *atp6*, *nad4* and *nad4l*, *nad6* and *cytb*) are fully conserved in the Callitettixini (Figure 1), and may represent a plesiomorphic conservative character for the insect mitogenomes [21]. They are assumed to improve the translation efficiencies of short PCGs such as *atp8, nad4l* and *nad6* [56], [57].

### Evolutionary patterns and estimation of the nucleotide substitution rate of the AT-rich region

Cook summarized the characters of the AT-rich region for arthropods and described the following general features: tandem repeat sequences, sequences of poly(T), a subregion with high A+T content, and stem-loop structures [58]. The AT-rich regions of all five studied Callitettixini species could be folded into the stem-loop structures (predicted by the mFOLD server).

The tandem repeat sequences are present in the mitogenomes of two (*A. contigua* and *C. braconoides*) of the five Callitettixini species studied here. The AT-rich region of *A. contigua* has a 515 bp tandem repeat area of 144 bp in length while in *C. braconoides* it is 588 bp in length with a 175 bp repeat unit (Table 3). No obvious tandem repeats are found in *A. producta* and *C. biformis*. In *C. versicolor*, however, the repeat sequences are present but not adjacent to each other. In contrast to that of the 5′ end, the repeat unit at the 3′ of the AT-rich region has a different nucleotide base and a 4 bp deletion. The tandem repeat sequences of both *A. contigua* and *C. braconoides* are located between the conserved segments 1 and 2. In *C. braconoide*, the 5′ part of the conserved segment 2 is shared with the 3′ end of the tandem repeat region, thus being also the part of the repeat unit. Tandem repeat sequences are present in *Philaenus spumarius* (Aphrophoridae) [26] but are absent in *Paphnutius ruficeps* (Cercopidae) [27]. It seems that the tandem repeat sequences may not be a plesiomorphic condition in *Paphnutius ruficeps* or Cercopidae. These differences within groups can also be observed in other hemipteran groups such as Heteroptera [16] or Fulgoromorpha [21]. The tandem repeat sequences might therefore not be good characters for phylogenetic inference.

**Table 3:** Nucleotide composition, total length and details on tandem repeats in the AT-rich regions of the five Callitettixini species.

	Nucleotide content (%)				Length(bp)	Tandem repeat	
	T	C	A	G		Length (bp)	Time*
*A. contigua*	40.2	11.3	41.6	6.9	1010.0	144	3.6
*A. product*	38.4	11.3	43.5	6.8	674.0	–	–
*C. versicolor*	37.5	12.5	44.0	5.9	775.0	–	–
*C. biformis*	35.3	12.2	44.0	8.4	629.0	–	–
*C. braconoides*	37.6	8.4	44.9	9.1	1040.0	175	3.3

*: the number of repeats.

**Figure 4:**
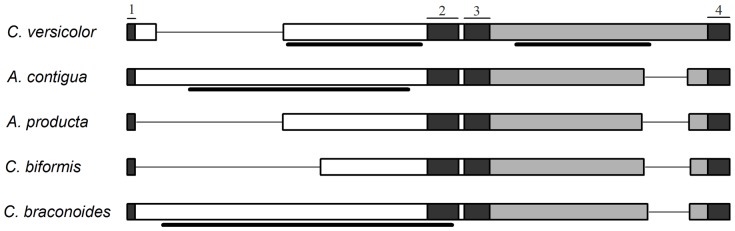
Schematic models of the AT-rich region and the relative position of the conserved segments. The black blocks indicate the conserved parts. The thick lines show the position of the repeat regions. The empty blocks indicate the none-conserved parts. The gray block represents the poly(N) area.

In the AT-rich regions of the mitogenomes of the five Callitettixini species, many Poly(N) sequences are found between the conserved segments 3 and 4, with the number of N ranging from five to nine. Except for the presence of the poly(T) sequences mentioned by Cook [58], poly(A), poly(C) and poly(G) re were also found in the AT-rich region in this study. In the AT-rich region of *C. versicolor*, two repeat regions are located at the 5′ and the 3′end of the conserved segment 4 (Figure 4).

**Figure 5:**
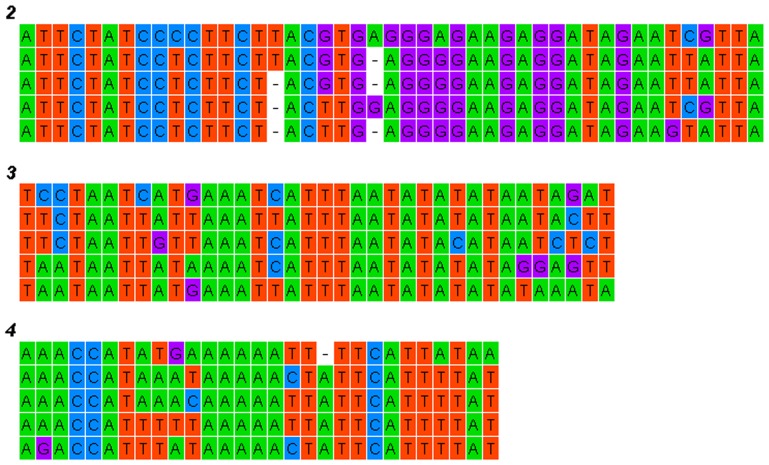
The sequences of the conserved area of the AT-rich regions of the five Callitettixini species. Numbers at the left-top corners are corresponding to the numbered conserved areas marked in figure 4.

**Figure 6:**
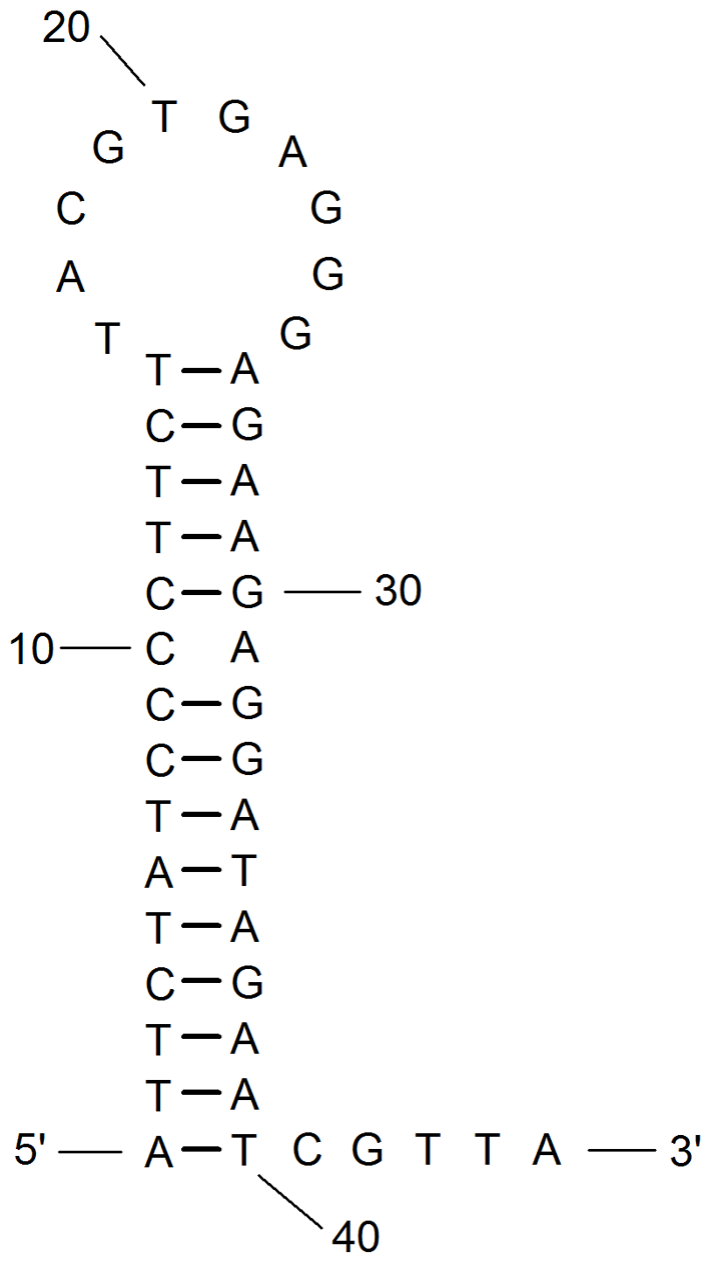
Putative secondary structure of conserved segment 2 (ΔG = −8.76 kcal/mol).

Calculating the rate of evolution in the AT-rich region is problematic for its low conservation in both sequence and organization [12], [53]. However, herein we found several short conserved segments within the five Callitettixini species. Models of the AT-rich regions are presented in Figure 4. In total, four conserved segments are found, with two shorter ones located at the two ends of the AT-rich region (Figure 4, marked as 1 and 4) and two longer ones located at the center of the AT-rich region (Figure 4, marked as 2 and 3). The conserved sequences are shown in Figure 5. It is notable that there are no homologous sequences in the other studied representative species of the Cicadomorpha [26], [27]. We therefore conclude that the presence of the conserved segments in the AT-rich region is a potential apomorphy for the spittlebug tribe Callitettixini. All of the four conserved segments could be folded into stem-loop structures (Figure 6) and all the stem-loop structures in the conserved segments 1, 3 and 4 are short while one stem-loop structure in segment 2 is long and stable (ΔG = −8.76 kcal/mol). Conserved segment 2 also has a fairly high (38.8%) GC content compared to the others. Next to these short conserved segments, homologous sequences which are longer than 50 bp were identified between two pairs of species (*C. biformis* + *C. braconoides* and *A. contigua* + *A. producta*) through dot-plot analyses. The positions of these homologous sequences are shown in Figure 4. In order to estimate the substitution rates (K2p distances) we aligned the AT-rich region and concluded that it evolves much faster than the other areas of the mitogenome (Table 4). The substitution rate of the AT-rich region is around 2–5 times faster than *atp8*, the fastest evolving gene of the mitogenome. This substitution rate has not been calculated before, so no comparisons to other species can be made.

**Table 4:** Pairwise distance (K2p) of the AT-rich region, *cox1* and *atp8.*

Pairs	K2p distances		
	AT-rich region	*cox1*	*atp8*
*C. braconoides*	0.318	0.112	0.173
*C. biformis*			
*A. contigua*	0.609	0.093	0.127
*A. producta*			

The AT-rich region is the one of the mysteries of the mitogenome for many reasons, including its biological function, great variation in length, motif patterns and nucleotide sequence difference. It is known that this region evolves faster than others in the mitogenome, but this has not been quantified. Prior to the present study, we attempted to estimate the substitution rates in higher taxa at different sampling ranges (for example, family, superfamily and order) but failed because no reliable sequence alignments could be obtained. In the present work we studied the Callitettixini at the level of tribe. No surprisingly, our results showed that there are significant differences in this region at this level. However, partial sequences of this region were found to have similarities in the mitogenomes of all five Callitettixini species we sequenced. It was these which permitted estimation of the evolutionary rate.

The AT-rich region is considered to play an important role in mitogenome DNA replication [12], [24]. The conserved sequences, stem-loop structures and tandem repeat sequences found in the present study can provide useful information for future research of the biological function of the AT-rich region. Another interesting question is how functionality is retained under such great variation both in length and sequence. Considering the high nucleotide substitution rate, both secondary structures and the conserved segments of the AT-rich region might be the key clue in understanding the function of the AT-rich region.

### Nucleotide substitution rates across the genes of the mitogenome

Nucleotide substitution rates (NSR) vary across different genes of the mitogenomes. In general, the PCGs evolve faster than the tRNA and rRNA genes. The average NSR of PCGs of the mitogenomes of the five Callitettixini species is 0.16, while for rRNAs and tRNAs, they are 0.09 and 0.093 respectively. The fastest evolving PCG is *atp8* (NSR = 0.237) and the slowest is cytochrome c oxidase subunit I (*cox1*) (NSR = 0.132). The J-chain PCGs and N-chain PCGs have the same average NSR. However, the tRNA genes encoded by the J-chain evolve slower than those encoded by the N-chain (NSR 0.074 vs 0.135).

The NSR for every codon position of each gene was calculated and is given in Table 5. As shown in the table, the third codon position has the highest total substitution rate (0.330), while the first and second codon positions show distinctly lower total rates (0.120 and 0.057, respectively). Additionally, the first and second codon positions show a much higher degree of variation among the different genes than the third position. These differences indicate that each gene and each codon position evolves under different constraints. The first and second positions show a lower evolutionary rate than the third since they are more essential in specifying amino acids. The same situation is observed between the second and the first codon positions. A similar correlation has also been reported for other metazoan mitogenomes [59]. On the amino acid level, the genes of cytochrome oxidase subunits I, II and III (*cox1*, *cox2*, and *cox3*) and cytochrome b (*cytb*) are the slowest evolving proteins, while the subunit of ATP8 is the fastest (Table 5). This is consistent with the differences of the nucleotide substitution rates among the PCGs.

**Table 5:** Substitution rate of the mitogenomes of the five Callitettixini species.

	Amino acid (PAM@)	Codon (K2p)					
		Total	Codon positions			Ti/Tv	Ka/Ks
			1	2	3		
*atp8*	0.408	0.237	0.176	0.141	0.434	0.8	0.3487
*atp6*	0.149	0.160	0.135	0.061	0.307	1.1	0.1679
*cox1*	0.041	0.132	0.076	0.011	0.355	1.7	0.0383
*cox2*	0.076	0.140	0.093	0.026	0.346	1.7	0.0578
*cox3*	0.118	0.152	0.101	0.049	0.342	1.5	0.1074
*Cytb*	0.139	0.165	0.116	0.039	0.385	1.1	0.1187
*nad2*	0.256	0.195	0.197	0.098	0.307	0.9	0.2930
*nad3*	0.179	0.164	0.123	0.074	0.320	0.9	0.2114
*nad6*	0.241	0.182	0.140	0.119	0.303	1.2	0.2599
**Total-J-PCG**	**0.139**	**0.160**	**0.119**	**0.052**	**0.340**	**1.2**	**0.1290**
*nad1*	0.163	0.150	0.105	0.045	0.339	1.2	0.1501
*nad4*	0.171	0.140	0.097	0.058	0.286	1.0	0.1993
*nad4l*	0.208	0.175	0.161	0.049	0.350	1.0	0.2572
*nad5*	0.235	0.177	0.147	0.084	0.321	0.9	0.2412
**Total-N-PCG**	**0.197**	**0.160**	**0.123**	**0.065**	**0.315**	**0.9**	0.2091
**Total-PCG**	**0.160**	**0.160**	**0.120**	**0.057**	**0.330**	**1.1**	0.1559
16S rRNA	-	0.089	-	-	-	0.8	-
12S rRNA	-	0.090	-	-	-	0.7	-
**Total-J-tRNA**	**-**	**0.074**	**-**	**-**	**-**	**0.7**	-
**Total-N-tRNA**	**-**	**0.135**	**-**	**-**	**-**	**1.33**	-
**Total- tRNA**	**-**	**0.093**	**-**	**-**	**-**	**0.953**	**-**
**Total genome** *	**-**	**0.142**	**-**	**-**	**-**	**0.9**	**-**

*: AT-rich region and other intergenic spacers not included;

@: calculated using Dayhoff matrix.

### Correlations between GC content and the substitution rate

**Figure 7:**
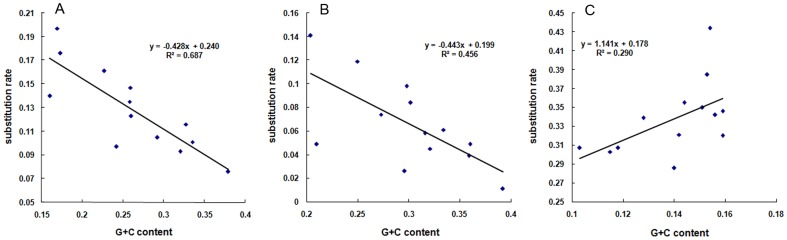
Correlations between the GC content and the substitution rate (K2p). The regression line and their equations are showed to visualize the trends. A: the 1st codon position; B: the 2nd codon position; C: the 3rd codon position.

When the GC content of the first and second codon positions is compared to the substitution rate, a clear negative correlation is observed (Figure 7). However, in the third codon position it is slightly positive (Figure 7). Ratios of the non-synonymous and synonymous substitutions (Ka/Ks) were calculated in order to compare the purifying pressures upon the different codon positions (Table 3). The Ka/Ks ratios for all PCGs are below 1.0, indicating that these genes evolved under purifying selections. The results indicate that the mitogenome genes are functionally constrained and that changes of amino acids are harmful to function. The relative stabilities of amino acid sequence are necessary for the organisms, and the observed differences in the accumulation of mutations at different codon positions are reflective of the pressure of purifying selections. The three codon positions are subject to different selection pressures during evolution and the GC content is positively correlated with the pressures. The tRNA and rRNA genes have a similar GC content and substitution rate (Table 2 and Table 5).

### Phylogenetic reconstruction

Based on the mitogenome data, the phylogenetic relationships of the six cercopoid species (including the five Callitettixini species (Cercopidae) and *P. spumarius* (Aphrophoridae)) were constructed with three additional Hemipteran species (*H. vitripennis*: NC_006899; *Saldula arsenjevi*: NC_012463.1; and *Nezara viridula*: NC_011755.1) as outgroups. The final data matrix contained a total of 15,071 nucleotide sites.

**Figure 8:**
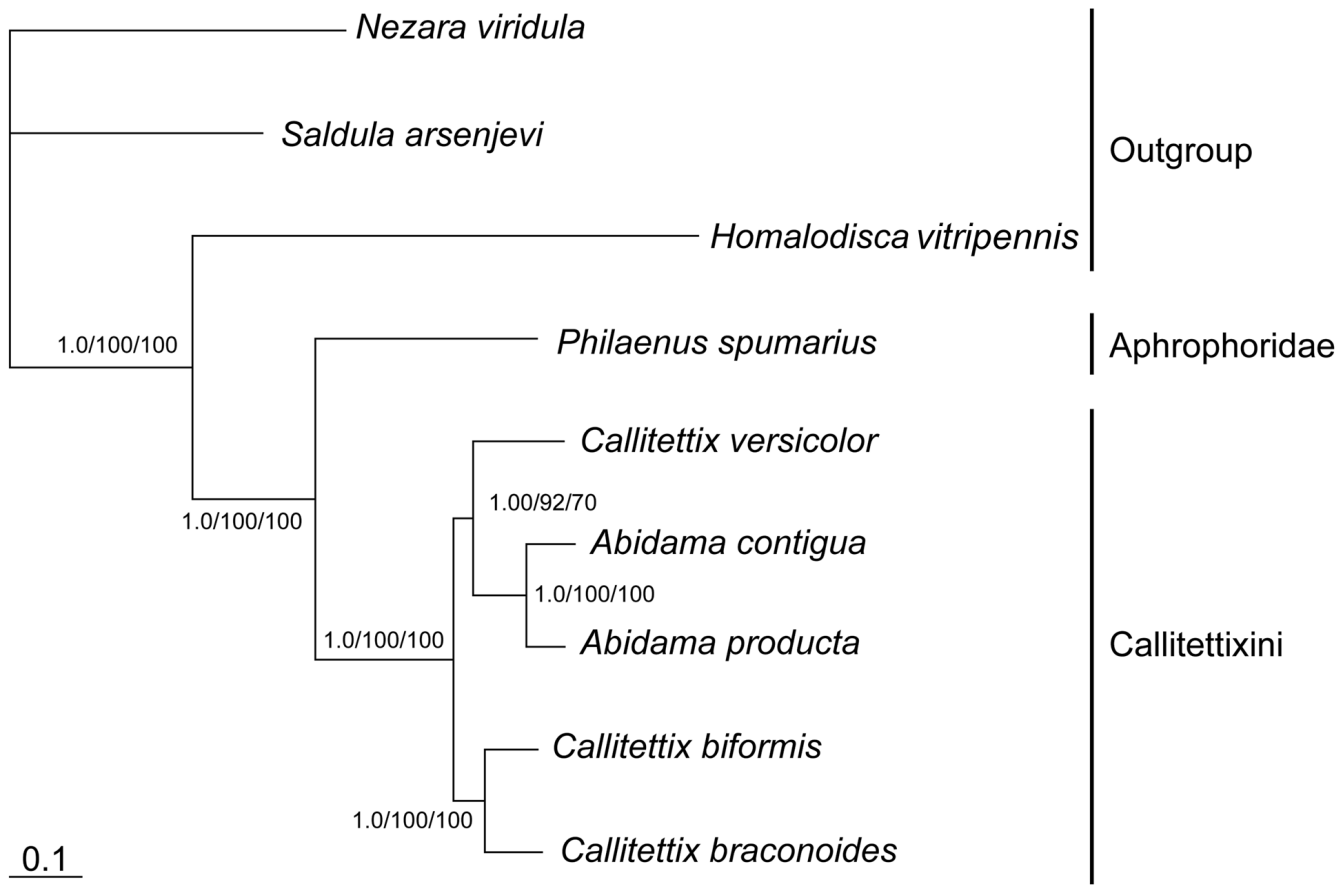
Relationships inferred from bayesian interface based on a 15071 nucleotide matrix (−LnL = 80211.79). Maximum likelihood (−LnL = −83131.48) and maximum parsimony analyses yield the same topology (tree length  = 16509, 6495 sites constant and 5058 sites informative). The numbers near the nodes give Bayesian posterior probability, the bootstrap values from pseudoreplicates of the maximum likelihood and maximum parsimony analyses.

The maximum likelihood, Bayesian and maximum parsimony analyses resulted in the same branching pattern as shown in Figure 8. All nodes of the tree are strongly supported by the bootstrap values and the bayesian posterior probability. The same topology was also retrieved in the analysis where the third codon position was excluded. Figure 8 is also supported by the phylogenetic analyses of the Callitettixini based on morphological data (Liang, in prep.).

The present study shows that the mitochondrial genomes might be good molecular markers for the phylogenetic inference at the species level. However, concerning the introgression of mitochondrial DNA into closely related species as reported in some vertebrates [60] and invertebrates [61], the use of mitochondrial DNA alone for phylogenetic analyses has the potential to mislead.

## Conclusions

In the present study, we analyzed the evolutionary characteristics of the mitogenomes of five species of the spittlebug tribe Callitettixini (Hemiptera: Cercopidae). The mitogenomes of these five species show an extreme AT bias (avg. 76.7%), and their gene arrangements are identical with the putative ancestral gene order of insects. The genes encoded evolved at different rates of substitution, with PCGs having a higher rate than the tRNA and rRNA genes. The *cox1*, *cox2*, *cox3 and cytb* are the slowest evolving genes while *atp8* is the fastest. In total, the substitution rates computed from our dataset (according to K2p distances) for the PCGs, tRNA genes and rRNA genes are 0.16, 0.09 and 0.093, respectively. In the PCGs, the first and second codon positions show slower rates than the third. The substitution rates are negatively correlated with the GC contents in the first and second codon positions but positive in the third. The AT-rich region is the most variable part of the mitogenome. Interestingly, four short conserved segments are also found within the mitogenomes of the Callitettixini species and these are not present in other Cicadomorphan species. The substitution rate of these conserved segments of the AT-rich region is 2–5 times faster than the *atp8*, the fastest evolving encoding gene of the mitogenomes.

The mitochondrial genome remains a useful and easily used molecular tool for addressing the evolutionary problems at different levels, such as genus, tribe and family. However, mitogenome data for the tribe level is still lacking (except for some model organisms). More work on more representative taxa is needed.

## Supporting Information

Figure S1 **Cloverleaf folds of the 22 tRNAs of the mitogenome of** ***Abidama producta*** **.**
(TIF)

Table S1 **Primers used in this study.**
(DOC)
