# Supplementary figures and images for: Comparative Analysis of the Mitochondrial Genomes of Callitettixini Spittlebugs (Hemiptera: Cercopidae) Confirms the Overall High Evolutionary Speed of the AT-Rich Region but Reveals the Presence of Short Conservative Elements at the Tribal Level
Source: PLoS One. 2014 Oct 6;9(10):e109140. doi: 10.1371/journal.pone.0109140 (PMC4186805; doi:10.1371/journal.pone.0109140)

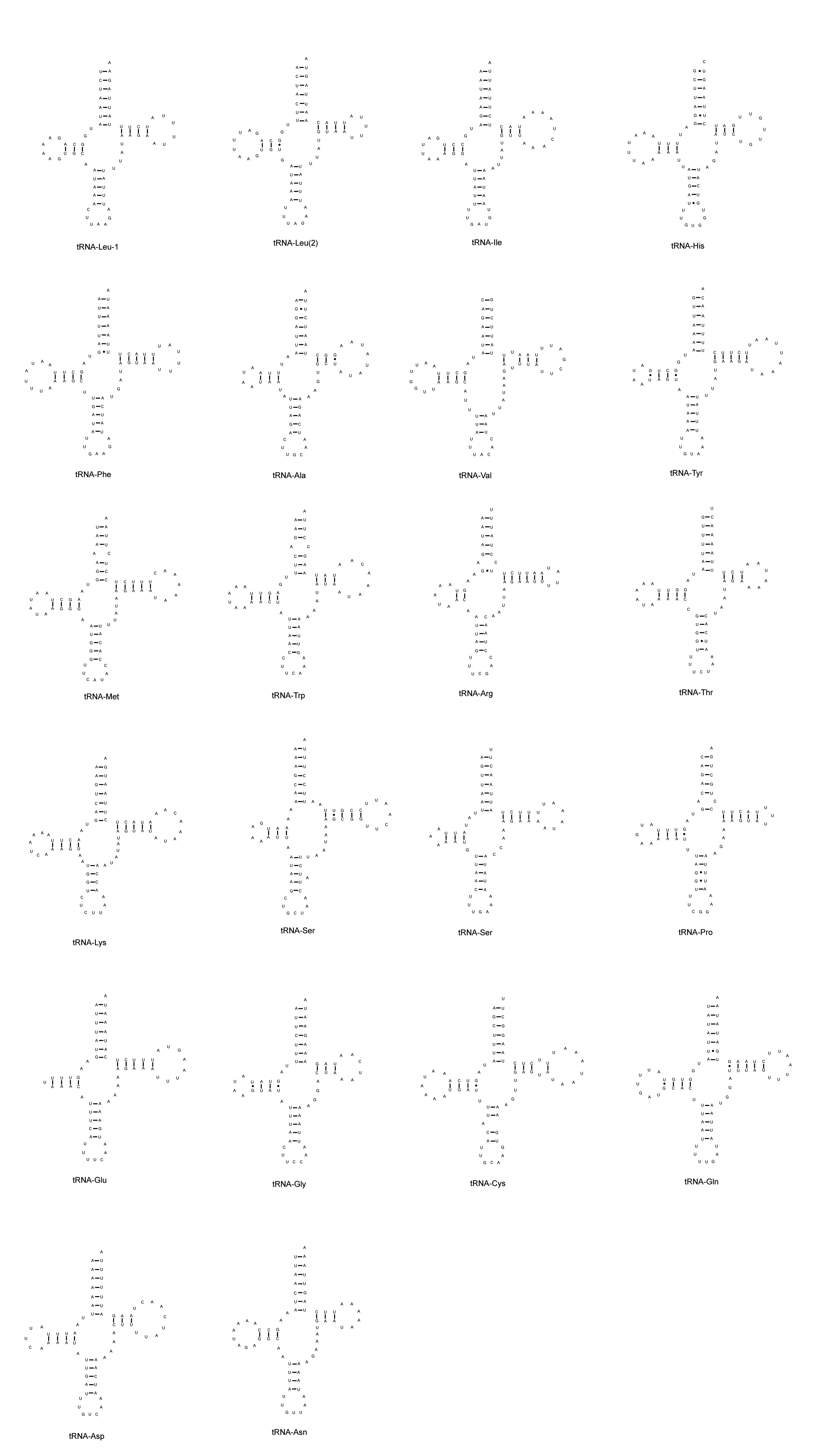

Supplement: Figure S1 — Cloverleaf folds of the 22 tRNAs of the mitogenome of Abidama producta . (TIF) [file pone.0109140.s001.tif]
