# Supplementary material for: Comparative Analysis of the Mitochondrial Genomes of Callitettixini Spittlebugs (Hemiptera: Cercopidae) Confirms the Overall High Evolutionary Speed of the AT-Rich Region but Reveals the Presence of Short Conservative Elements at the Tribal Level
Source: PLoS One. 2014 Oct 6;9(10):e109140. doi: 10.1371/journal.pone.0109140 (PMC4186805; doi:10.1371/journal.pone.0109140)
Supplement: Table S1 — Primers used in this study. (DOC) [file pone.0109140.s002.doc]

Table S1 Primers used in this study

| Name | Sequences(5'-3') |
| --- | --- |
| 10611S | CCTACCATGAGGTCAAATATC |
| 10715A | CCTCCTCAAATTCATTTTACTA |
| 10XXS | GGATTACCTCCTATACTAGGATTTT |
| 11520S | ATCATAACGATAACGAGGTAA |
| 11753A | GATTTTGCTGAAGGTGAATC |
| 12842A | CCTTTGCACAGTCAGAATAC |
| 13662S | TCAAATTAAATTGAATTGCACAA |
| 14088S | ACCGCCAAATTCTTTGAAT |
| 169A | AATARGGTATGAACCYATTAGCTT |
| 24XXA | AATGGCYAATACTGCTCCT |
| 3272S | AAAWCWATTGGACATCAATGATA |
| 3563S | CCACAAATTTCTGAACATTG |
| 4217S | GATCAAGACACCTAGTATTTACACT |
| 4440A | ATGWCCWGCAATTATATTWGC |
| 5007S | AATCARGATATYGGTTTTATAGG |
| 5337A | CATCAACAAAATGTCAGTATCA |
| 6160A | TCAATTTTRTCATTAACAGTGA |
| 6165A | AAAGCATAAYATTGAAGAT |
| 6981A | TTAARGCTTTATTATTTWTATGTGC |
| 7295S | AAAGGGTAATTGAGCTCTCTTAGT |
| 7701A | CATTTGTWTTAGGTATTTCWTC |
| 8661S | GGAGCTTCWACATGAGCTTT |
| 8680A | AAAGCTCATGTWGAAGCTCC |
| 9S | CCTGATTAAAGGATTATTTTGATGT |
| C1N2353 | GCTCGTGTATCAACGTCTATWCC |
| C3N4908 | CGAGTTAYATCTCGTCATCATTG |
| C3N5460 | TCAACAAAATGTCARTAYCA |
| C6C5587A | TTAATTGGATCAAATCCACATT |
| C6N5988S | ACTAAAGCCAAAATAGAGGCAAT |
| C6N7033A | AATCAGGATATTCGTTTTATAGG |
| DM4885S | CATTYTTTTGAAGATTYTTTCATA |
| DM5892A | ATTCTTAAWTWTTAGGTCGAAACT |
| DM2-10XXS | AGGWCTTCCACCTATAYTAGGATT |
| DM5916S | TTCCTATTTAATTAACTAAAGCC |
| DM7514A | TARGTCTTWTAATGCTGGWATA |
| DM8463S | GAAGAATAAGCAATTAAAGACTT |
| DM9583A | ATGATTTACAAAACCATTGTT |
| DMYX4274S | AGCAYTACCAATATGAWTATYATT |
| LRN13000 | TTACCTTAGGGATAACAGCGTAA |
| N3J5747 | CCATT TGAAT GTGGR TTTGA YCC |
| N4LN9629 | GTTTGTGAGGGWGYTTTRGG |
| N5J7077 | TTAAATCCTTWGARTAAAAYCC |
| P1612A | TTGYGATAAGTCGTAACAAAGTA |
| P1612S | CGGTYTGAACTCAGATCATGT |
| PC12A | CAAATTTCTGAACAYTGACCA |
| PC12S | TGAGCTCATCATATATTTACTGT |
| SRN14745 | GTGCCAGCAGYYGCGGTTANAC |
| TWJ1301 | GTTAAWTAAACTAATARCCTTCAAA |
| TWN1284 | ACARCTTTGAAGGYTAWTAGTTT |

Note: including the primers that specific to each species
